# Progression Risk in Cancer Patients with HIV Compared with Cancer Patients Without HIV

**DOI:** 10.1093/cid/ciag170

**Published:** 2026-03-09

**Authors:** Derek Schneider, Nathan W Van Bibber, Jessica Y Islam, Anna E Coghill, Gita Suneja

**Affiliations:** The Warren Alpert Medical School of Brown University, Providence, Rhode Island, USA; Cancer Epidemiology Program, H. Lee Moffitt Cancer Center and Research Institute, Tampa, Florida, USA; Cancer Epidemiology Program, H. Lee Moffitt Cancer Center and Research Institute, Tampa, Florida, USA; Cancer Epidemiology Program, H. Lee Moffitt Cancer Center and Research Institute, Tampa, Florida, USA; Huntsman Cancer Institute, The University of Utah, Salt Lake City, Utah, USA; Department of Radiation Oncology, The University of Utah Spences Fox Eccles School of Medicine, University of Utah, Salt Lake City, Utah, USA

**Keywords:** HIV (human immunodeficiency virus), cancer progression, oncology outcomes, non-AIDS-defining cancers, health disparities

## Abstract

In a retrospective observational study investigating time to cancer progression and death in people with and without human immunodeficiency virus (HIV), an HIV diagnosis was associated with elevated risk of cancer progression compared with people without HIV. Accelerated cancer progression may contribute to the observed cancer survival disparity for people with HIV.

## Graphical Abstract

**Graphical Abstract:**
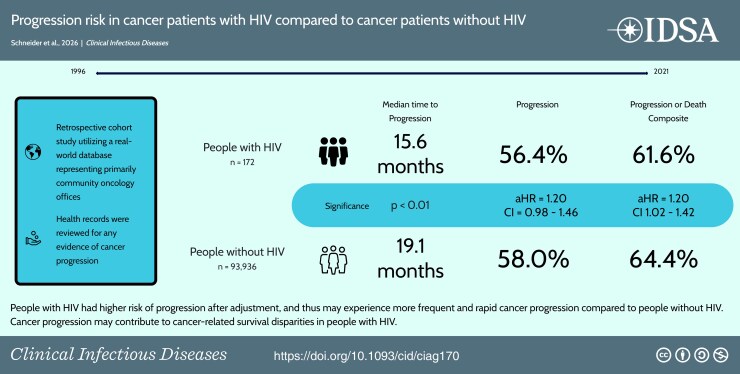
This graphical abstract is also available at Tidbit: https://tidbitapp.io/institutional-portal/clinical-infectious-diseases/tidbits/progression-risk-in-cancer-patients-with-hiv-compared-to-cancer-patients-without-hiv-b38bef30-d73c-425d-b5aa-b74898879f98/update.

HIV is associated with an increased risk for several common cancers and an elevated risk of cancer-specific mortality after a cancer diagnosis [1–4]. After controlling for clinical and demographic factors that influence cancer survival, such as advanced cancer stage at diagnosis, insurance status, or delayed cancer treatment, the survival deficit for people with HIV (PWH) persists for multiple cancers [2, 5–7].

Despite data consistently demonstrating a cancer survival disparity for PWH, research examining cancer progression as one potential underlying driver of this poor survival is lacking. Given the altered immune environment in which cancers develop among PWH, an accelerated disease process (eg, progression after initial cancer treatment) might drive the survival disparity [8]. In this study, we evaluated the impact of HIV on cancer progression and survival using the Flatiron Health database. We hypothesized that cancer progression risk would be elevated in cancer patients with HIV compared with those without HIV.

## METHODS

We used electronic health record (EHR)-derived data from 1996 through 2021 from the Flatiron Health database. The Flatiron Health database is a longitudinal, real-world database comprised of deidentified, patient-level EHR data from approximately 280 cancer clinics (800 cancer care sites) across the US [9, 10]. Most patients in the database originate from community oncology settings; relative community/academic proportions may vary depending on the study cohort. The data were subject to confidentiality protections to prevent reidentification.

The following International Classification of Diseases (ICD) codes were used to select PWH: 42, 42.1, 42.2, 42.9, 43, 43.1, 43.2, 43.3, 43.9, 44, 44.9, 79.53, 795.8, B20, B97.35, O98.72, O98.71, O98.7. Those without an HIV diagnosis code were considered people without HIV (PWoH). The following cancer cohorts from the Flatiron data warehouse were evaluated in this study: advanced melanoma, gastric carcinoma, hepatocellular carcinoma (HCC), metastatic renal cell carcinoma, metastatic breast cancer, multiple myeloma, nonsmall cell lung cancer (NSCLC), and small cell lung cancer (SCLC).

The primary outcome was time to cancer progression, which was defined as the number of days from cancer diagnosis to the date of real-world progression (rwP). Flatiron Health defines rwP as any distinct episodes of clinician-described disease progression in the medical record, which may include radiographic findings, pathology reports, or clinician described disease progression in the disease of interest [11]. Follow-up for cancer progression was limited to the date range with confirmed medical record availability (ie, days from cancer diagnosis to last clinical note) for each patient, given that the outcome of rwP was assessed directly through medical record abstraction. Approximately 3.6% (*N* = 3404 [3396 PWoH, and 8 PWH]) of patients had a first clinical note listed after diagnosis but were missing further clinical note details and were therefore excluded from rwP assessment. A secondary endpoint was time to progression or death, which was defined as the days from cancer diagnosis to the earliest date of either rwP or death during the above-defined follow-up period. If neither rwP nor death were observed in the Flatiron Health dataset, indicative of a patient still alive and under cohort follow-up, the last date was right censored to the final date of the later of the last clinical note or the date of the most recent Flatiron data pull for this analysis (15 February 2021).

Times to cancer progression and to the combined progression or death outcome were each compared among cancer patients with and without HIV by plotting Kaplan-Meier survival curves. We estimated adjusted hazard ratios (aHR) with 95% CIs (95% CI) using cox proportional hazards regression, adjusting for sex, race, age at cancer diagnosis, smoking history (yes/no), and log-transformed time to initiation of cancer therapy after cohort entry. Patients who progressed after cancer diagnosis but prior to initiation of any cancer treatment were not eligible for inclusion.

## RESULTS

This study included 94 108 cancer patients, 172 of whom had evidence of HIV in their medical record (Supplementary Table 1). PWH were more likely to be male (69.2% vs 45.3%), non-White (60.5% vs 32.7%), and were on average younger at cancer diagnosis (58.9 vs 65.3 years) compared with PWoH. The most common cancer for both PWH and PWoH was NSCLC (PWH: 48.8%, PWoH: 47.7%), followed by HCC (13.4%) and gastric cancer (10.5%) in PWH, and breast cancer (20.8%) and multiple myeloma (8.3%) in PWoH.

**Figure 1.**
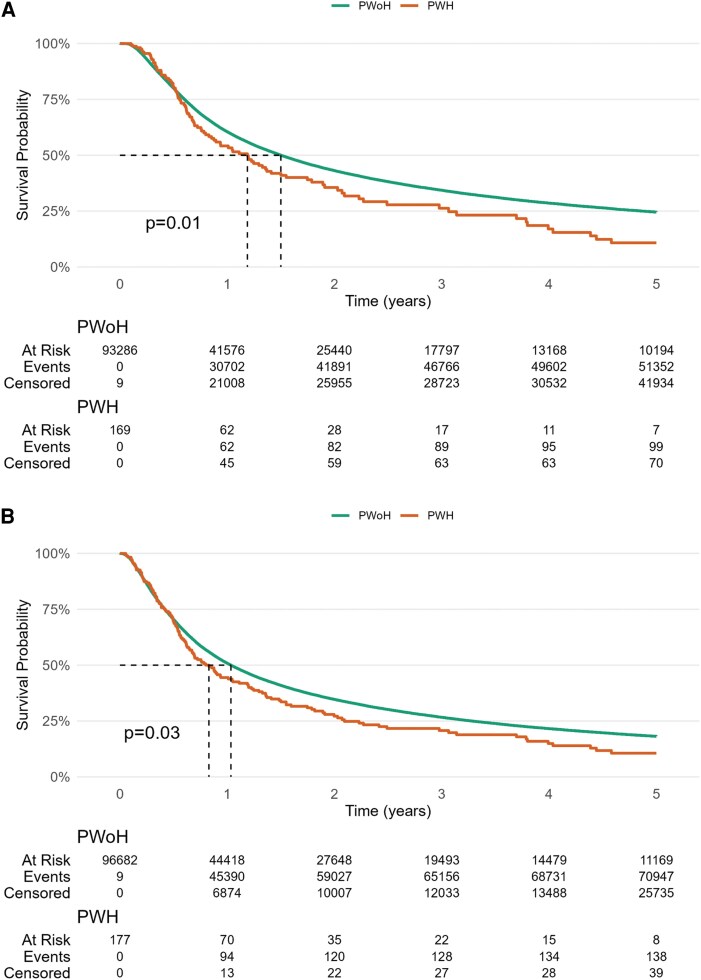
Kaplan-Meier curves representing time from cancer diagnosis to (*A*), real-world cancer progression (rwP), and (*B*), rwP or death, during the first 5 years following cancer diagnosis. Unadjusted *P* values were generated using the log-rank test specific to the first 5 years after a cancer diagnosis.

Fifty-eight percent of study patients had evidence of rwP in the EHR, including 56.4% of PWH and 58.0% of PWoH. PWH experienced significantly shorter time to rwP after cancer diagnosis compared with PWoH (50% progression probability of 15.6 months [PWH] vs 19.1 months [PWoH]; *P* < .01). Figure 1*A* demonstrates the 5-year real-world progression-free (rwPF) probability by HIV status. On multivariable analyses, HIV was associated with a 20% increased risk of rwP over the full course of study follow-up (aHR = 1.20; 95% CI = .98–1.46; *P*-value = .08).

We observed that 64.4% of study patients were recorded as deceased (61.6% PWH; 64.4% PWoH) during study follow-up. For patients with rwP, 73% were deceased, with an average time from date of progression to date of death among the deceased patients of 7.2 months versus 10.9 months for PWH versus PWoH, respectively. Figure 1*B* demonstrates the 5-year real-world progression-free/survival (rwPFS) probability by HIV status. HIV was associated with a statistically significant 20% increased risk of the combined outcome of either rwP or death over the full course of study follow-up (aHR = 1.20; 95% CI = 1.02–1.42; *P*-value = .03). These findings were consistent when limiting the data to the modern HIV treatment era (2005–2021).

Patient characteristics and HIV-associated hazard ratios for survival, progression, and the combined progression or death outcome for specific cancer sites with >10 cases among PWH (NSCLC, HCC, gastric carcinoma, breast cancer, and multiple myeloma) are summarized in Supplementary Table 2.

## DISCUSSION

We hypothesized that PWH and cancer would experience a higher risk of cancer progression after diagnosis compared with cancer patients without HIV. Using a dataset with collated real-world cancer treatment and progression data from medical records at >280 cancer clinics across the US, we observed a shorter duration to cancer progression among PWH diagnosed with advanced stage cancers and a statistically significantly higher risk of the combined outcome of cancer progression or death after adjustment for age, sex, race, smoking status, and time to initiation of cancer therapy after cohort entry. To our knowledge, this study is the first to demonstrate potential evidence of a reduced time to cancer progression in PWH receiving cancer treatment for advanced stage disease using real-world EHR data.

A prior study of PWH using SEER-Medicare claims-based data focused on cancer patients ≥65 years of age reported higher rates of death or retreatment (ie, claims for additional cancer therapy lines used as a proxy for relapse) for breast (*N* = 50) and colorectal cancer (*N* = 34) among PWH [4]. The current analysis expanded the range of cancers evaluated and directly assessed cancer progression from the medical record, an improvement over the claims-based definition. Our findings provide additional evidence that more rapid disease progression can occur in PWH and cancer; this may be one of the drivers of poor survival in PWH and cancer that we and others have consistently reported.

Most patients in the Flatiron Health database have advanced cancer, as the data are purposefully sourced from patients in community medical oncology clinics with a diagnosis of advanced cancer (89.1% of patients were stage IV), potentially limiting generalizability to earlier stages of disease [12]. As advanced cancers behave more aggressively, HIV-associated differences in time to cancer progression may be more evident in earlier-stage disease, a possibility that should be explored in future work. Because Flatiron Health includes EHR-abstracted data, there is the potential for misclassification of the rwP outcome assessed; however, we would not expect misclassification to be differential by HIV status as abstractions were conducted uniformly across the cohort. In addition, there is limited data on intensity of chemotherapy administered, for example number of cycles of specific agents, however we were able to determine that there were no significant differences by HIV status in the number of lines of systemic therapy administered or the duration of systemic therapy for NSCLC, HCC, or gastric carcinoma.

In conclusion, this study compared progression after an advanced stage cancer diagnosis between PWH and PWoH and observed evidence suggesting that PWH may experience more frequent and rapid cancer progression compared with cancer patients without HIV in the real-world setting. This association could contribute to a cancer survival disparity for PWH.

## Supplementary Material

ciag170_Supplementary_Data

## Supplementary Data

Supplementary materials are available at *Clinical Infectious Diseases* online. Consisting of data provided by the authors to benefit the reader, the posted materials are not copyedited and are the sole responsibility of the authors, so questions or comments should be addressed to the corresponding author.
